# Proof of principle: Physiological transfer of small numbers of bacteria from mother to fetus in late-gestation pregnant sheep

**DOI:** 10.1371/journal.pone.0217211

**Published:** 2019-06-06

**Authors:** Kevin Yu, Michelle D. Rodriguez, Zubin Paul, Elizabeth Gordon, Kelly Rice, Eric W. Triplett, Maureen Keller-Wood, Charles E. Wood

**Affiliations:** 1 Department of Physiology and Functional Genomics, University of Florida College of Medicine, Gainesville, Florida, United States of America; 2 Department of Microbiology and Cell Science, University of Florida Institute of Food and Agricultural Sciences, Gainesville, Florida, United States of America; 3 Department of Pharmacodynamics, University of Florida College of Pharmacy, Gainesville, Florida, United States of America; Leibniz Institute for Zoo and Wildlife Research (IZW), GERMANY

## Abstract

Fetal development is thought to proceed in a sterile environment. Recent reports of the presence of bacterial DNA in human placenta, the transfer of live bacteria from mother to fetus after hypoxia in the pregnant sheep, and the presence of bacteria in the meconium of newborn infants have suggested that the fetus might be exposed to bacteria *in utero*. The present experiments were designed to test the hypothesis that small numbers of bacteria introduced into the maternal bloodstream (too few to induce fever or changes in maternal food consumption), can be found in the fetus days later. We injected 100 colony forming units of green-, red- and far red- fluorescent protein (GFP, RFP, FRFP) expressing *S*. *aureus* into late-gestation pregnant sheep intravenously. Five to 7 days later, the animals were euthanized and tissues collected for analysis of GFP. The inoculations did not cause any fever or other measurable behavioral response in the ewes, but did result in the appearance of GFP DNA, and protein in various tissues within the fetuses. Immunohistochemical analysis reveals GFP protein-containing bacteria that appear to be mostly contained within other cells. We were unable to recover any live GFP-expressing bacteria from the fetal tissues. We conclude that *S*. *aureus*, and perhaps other bacteria, gain access to the fetus, although it is not clear from these experiments that they survive in the fetus. It is possible that these low inocula and their progeny were effectively cleared by the fetal immune system.

## Introduction

The fetus is thought to develop in a sterile environment [1]. The presence of bacteria within the feto-placental unit is thought to represent infection–associated with a risk of premature labor [2, 3]. In recent experiments, we have discovered that transient hypoxia in pregnant sheep caused an influx of small numbers of bacteria from mother to fetus [4]; associated with that influx was a transcriptomics signature of increased activity of inflammation pathways in fetal brain [5] and kidney [6]. While the presence of bacterial DNA [7] and histological evidence of bacteria [8, 9] in human placenta has been reported, the concept that there could be live bacteria transferred to the fetal compartment is debated [10]. In our own experiments [4], we used whole genome sequencing to prove live bacteria isolated from the placenta and fetal brain after hypoxia were identical, suggesting that there is transfer of bacteria from placenta to the fetus under the right conditions. While our data strongly suggest the transfer of live bacteria from mother to fetus, our previous experiments did not provide proof of this concept. We designed the present study to directly test the hypothesis that bacteria in maternal blood can cross the maternal/fetal barrier and into the fetus.

In the present experiments, we used *Staphylococcus aureus* bacteria labeled with plasmids encoding fluorescent proteins to provide proof of transfer of bacteria from mother to fetus. As we have previously reported, each bacterium received a GFP, RFP, or FP650 plasmid that replicates upon bacterial replication [11]. The plasmids in these fluorescence labeled bacteria do not appear naturally in sheep and are therefore absent in sheep not inoculated with these specific organisms. We used these bacteria to test the hypothesis that injection of bacteria into the maternal blood results in the transfer of those bacteria to the fetus.

## Materials and methods

### Animals

These experiments were approved by the University of Florida Animal Use and Care Committee. All experiments and procedures were performed in accordance with approved guidelines and regulations. All time-dated pregnant sheep were purchased from Advanced Ovine Solutions (Attica, NY, USA). We performed in vivo experimentation on 7 pregnant sheep of mixed western breeds carrying twin pregnancies (14 fetuses). We analyzed tissues from these fetuses and from banked tissues from an additional 32 fetuses of similar gestational ages. The banked tissues were used for quality control (“blank” values) in our PCR and ELISA quantification for fluorescent protein plasmid DNA and protein and were from both singleton and twin pregnancies.

### Staphylococcus aureus

The labelling and production of fluorescent protein-expressing *S*. *aureus* used in this study has been described previously [11]. Briefly, we constructed reporter plasmids containing Green Fluorescent Protein (GFP), Red Fluorescent Protein (RFP), and Far Red Fluorescent Protein (FRFP) and inserted these plasmids into *Staphylococcus aureus* strain SH1000. We have reported that these resulting GFP, RFP, and FRFP-containing *S*. *aureus* were stable in vitro, and that when injected into pregnant sheep could be recovered live (as assessed by culture) from maternal liver and placenta [11]. In those initial experiments, GFP immunoreactive protein was identified in maternal liver by immunohistochemistry [11].

### Preparation of inoculants

Overnight cultures of *S*. *aureus* containing pSGFPS1, pSRFPS1, and pSFRFPS1 fluorescent protein-encoding plasmids were propagated in 10 mL of fresh TSA supplemented with 10 ug/mL trimethoprim until OD of ~ 0.4 was reached. Cultures were serially diluted to 100 cfu/mL in sterile 1X PBS as described previously [4]. Samples were kept on ice before *in vivo* inoculation to prevent excess growth beyond the desired concentration.

### Inoculation

Seven ewes, each carrying twin ovine fetuses, were inoculated with fluorescent protein-labeled *S*. *aureus*. Inoculum consisted of 100 cfu (colony forming units) each of GFP-, RFP-, and FP-650- expressing *S*. *aureus*. Inoculations were performed as transcutaneous injections into the external jugular vein of the ewe between 128–132 days of gestation. Each ewe therefore received a total of 300 cfu of fluorescent protein-labelled bacteria (100 cfu each of GFP-, RFP-, and FRFP- containing SH1000 *S*. *aureus*). Animals were allowed free access to food and water. Food intake was monitored and recorded daily. Sheep were not subjected to surgery or other substantially painful procedures and did not display any otherwise clinically significant signs of pain or discomfort, so were not treated with analgesics for pain control. Blood samples (10 mL) were taken once daily by direct venipuncture of the external jugular vein. Rectal temperatures were taken twice daily. Blood samples were examined as smears for the presence of fluorescent bacteria. Ewes were euthanized after 5–7 days and tissue samples were collected at necropsy. Euthanasia was performed using a single intravenous injection of a mixture of pentobarbital/phenytoin (at least 80 mg/kg pentobarbital and 1 mg/kg phenytoin sodium; Virbac Animal Health, Fort Worth, TX, USA) into the external jugular vein in accordance with the American Veterinary Medical Association Guidelines for Euthanasia of Animals (https://www.avma.org/KB/Policies/Pages/Euthanasia-Guidelines.aspx). Following cardiac arrest, bilateral thoracotomy was performed. Three samples of each tissue from each fetus was harvested, one set for immunohistochemistry, one set for DNA/RNA extraction, and one set for culture. Tissues collected for immunohistochemistry were fixed in 4% paraformaldehyde overnight, then transferred to 70% reagent alcohol in water until processed for paraffin embedding. Tissues collected for molecular analysis were immediately flash frozen in liquid nitrogen. Frozen samples were stored in -80°C until further processing. Tissues collected for culturing were chilled in sterile tubes on wet ice and promptly subjected to homogenization and culture.

In addition to the 100 cfu inoculation experiments, we performed several pilot experiments to guide our decision regarding inoculum dose. We inoculated one animal each with 10^4^, 10^6^, or 10^8^ cfu, but decided against using these doses in favor of a much lower dose, 10^2^ cfu, which did not cause any change in maternal body temperature or behavior, and which was not fetotoxic.

### DNA extraction and PCR

*1*00-150mg of tissue samples were set in 500μL lysis buffer (0.1M Tris-HCl:0.1M NaCl:0.005M EDTA:2%SDS) and 1mg/mL protease K and left to lyse for 3 hours with periodic vortexing. DNA was separated from the lysate using an equal volume of Phenol:Chloroform:Isoamyl Alcohol 25:24:1 Saturated with 10 mM Tris, pH 8.0, 1 mM EDTA (Sigma-Aldrich) and centrifugation at 12,000 RPM. The aqueous phase was mixed with 100% isopropanol to precipitate the DNA and mixed with 70% ethanol to remove residual proteins and carbohydrates. The DNA pellet was spun down at 12k RPM for 10 minutes and left to dry for 15 minutes. The pellet was then dissolved in 100-150mL ddH_2_O. PCR was performed using primers designed by PrimerBlast (Table 1).

**Table 1:** Primer sequences used for PCR amplification and detection of plasmids encoding fluorescent proteins.

FP	strand	Sequence (5'->3')	Template strand	Length	Start	Stop	T_m_	GC%	Amplicon Length BP
**GFP**	Forward	GCCACAACGTTGAAGATGGT	Plus	20	509	528	59.05	50	148
	Reverse	TGGTCACGCTTTTCGTTAGGA	Minus	21	656	636	59.93	47.6	
**RFP**	Forward	GGTCCATTACCATTTGCATGGG	Plus	22	182	203	59.9	50	513
	Reverse	ATGATGACGACCTTCTGTACGTT	Minus	23	694	672	59.81	43.5	
**FRFP**	Forward	GCTGATTCTGGTTTACGTGGTC	Plus	22	485	506	59.58	50	212
	Reverse	CGAGCTACAGCCATTTCATGT	Minus	21	696	676	58.72	47.6	

Using endpoint PCR, we tested for the presence of GFP, RFP, and FRFP plasmid DNA in fetal liver, spleen, placentome, and cerebral cortex in all 14 fetuses (Table 2). To formally test for possible false positive detection of GFP, RFP, or FRFP plasmid DNA, we included a parallel analysis of DNA extracted from tissues from an additional 7–14 fetuses from pregnancies that were not subjected to inoculation with fluorescent protein-encoding plasmid. All PCR products were electrophoresed in agarose gel to confirm correct product length. The specificity of the PCR reaction was confirmed for all primer sets by Sanger sequencing of the amplicons. Reactions producing amplicons of incorrect size were thrown out and not included in the results or used as evidence of presence of the plasmid. Chi-square statistics were used to test the null hypothesis of false detection.

**Table 2:** Daily food and hay consumption and rectal temperature data from sheep inoculated with fluorescent protein-expressing SH1000 *S*. *aureus*. Rectal temperatures in Fahrenheit during experimental period after inoculation (n = 7). Criterion for statistical significant was P<0.05 (One-way ANOVA). Data are represented by means ±SEM. No significant change was found.

Measured variable	Day 1	Day 2	Day 3	Day 4	Day 5	Day 6
**Pelleted food (grams)**	2186±40	2214±40	2229±47	2229±47	2214±40	2200±100
**Hay (grams)**	483±48	563±71	513±110	501±69	334±81	400±0
**Rectal Temperature**	101.8±0.2	101.9±0.1	102.1±0.2	102.3±0.2	102.0±0.2	102±0.4

### Immunohistochemistry

Tissue samples were immediately fixed using 4% paraformaldehyde overnight after necropsy. Tissue samples were transferred to 70% Reagent Alcohol for storage. Tissues were trimmed and embedded in paraffin as previously described [12]. Histological samples were cut to 5–7 **μ**m thickness using a Microm HM 325 (Thermo Fisher Scientific, Kalamazoo, MI) and mounted on slides. Nonspecific binding was blocked using 2.5% horse serum for 30 minutes. Specific GFP immunoreactivity was probed using polyclonal anti-GFP antibody (Millipore AB3080, MilliporeSigma, Burlington, MA) at a 1:500 dilution (2 **μ**g/ml) or monoclonal anti-GFP antibody (ThermoFisher MA5-15256) at 1:200 dilution. Specific staining of GFP immunoreactivity was visualized using two methods. First, by binding of a universal (anti-rabbit and anti-mouse) biotinylated antibody provided in the RTU Vectastain kit (Lot VEZ0402, Vector Laboratories, Burlingame, CA); DAB substrate was used for visualization of the anti-GFP antibody, and slides were counterstained with methyl green. The second method of visualization was immunofluorescence, accomplished by binding of monoclonal anti-GFP (ThermoFisher MA5-15256) primary antibody with VRDye 549 goat anti-mouse IgG (Part #926–54110, LiCor Corp., Lincoln, NE) or polyclonal anti-GFP (Millipore AB3080) primary antibody with VRDye 490 goat anti-rabbit IgG (Part #926–49120, LiCor Corp., Lincoln, NE). *S*. *aureus* was immunostained using monoclonal anti-*S aureus* (Abcam catalog number ab37644) at a 1:500 dilution and visualized using VRDye 549 goat anti-mouse IgG. Double immunostaining of GFP and *S aureus* was accomplished using polyclonal anti-GFP (Millipore AB3080) and monoclonal anti-*S aureus* (Abcam ab37644) in dilutions and with second antibodies as described above. Immunostained sections were coverslipped with Fluoromount containing DAPI for visualization of cell nuclei (Southern Biotechnology Associates, Birmingham, AL). Images were recorded at 10x and 40x zoom using Olympus BX41 microscope using both epifluorescence and bright field capabilities (Olympus Life Science, Tokyo, Japan).

### Culturing of fluorescent bacteria in tissue homogenates

Fetal liver, lung, spleen, and maternal placenta were tested for the presence of live *S*. *aureus* by classical culturing on BHI (Brain Heart Infusion) and TSA (Tryptic Soy Agar) medium supplemented with 10 ug/mL trimethoprim to select for fluorescent plasmids as described previously [11]. Approximately 50 mg of sample was mechanically homogenized in 500 uL of sterile 1x PBS solution, and serially diluted in a 1:10 ratio before spread-plating 100 uL of each dilution. All plates were incubated at 37C for approximately 4 days to ensure ample time for growth. On the following day, and plates were screened for the presence of colonies. To assess fluorescence, images were taken at 60X magnification using Olympus Digital Fluorescence Microscopy (Olympus Life Science).

### GFP ELISA

GFP ELISA was performed using the GFP SimpleStep ELISA kit (Abcam #ab171581, Cambridge, UK). Approximately 200mg frozen tissue samples were set in 600mL chilled cell extraction buffer and homogenized. Tubes were incubated on ice for 20 minutes then centrifuged at 18,000 x g for 20 minutes at 4°C. 500**μ**L supernatants were transferred to clean tubes and pellets discarded. 1.5mL acetone was added to the supernatant to precipitate the protein. Acetone was then removed by centrifugation. The pellet was then dissolved in 150**μ**L cell extraction buffer and assayed.

## Results

### Physiological response to inoculation

None of the ewes injected with 300 cfu of bacteria showed any sign of fever or altered behavior after inoculation. Body temperatures and food consumption were unaltered after intravenous inoculation of sheep with 100 cfu each of GFP, RFP, and FP650-labelled *S*. *aureus* (Table 2). All measured temperatures were within the normal range of body temperature for sheep (100.9–103.8° F) [13]. We were unable to detect fluorescent bacteria in the maternal blood samples, suggesting that the bacteria had cleared the maternal bloodstream within 24 hours of inoculation.

### Detection of plasmid DNA

We could detect GFP plasmid DNA in all fetal spleen, liver, and brain (cerebral cortex) samples analyzed (Table 3A). We were successful in detecting GFP plasmid DNA in 13 of the 14 fetal placentome samples (7/7 pregnancies). (In contrast, we were unable to detect GFP plasmid in any liver (n = 14), cerebral cortex (n = 7), placentome (n = 7), or spleen (n = 8) samples from fetal sheep whose mothers were not inoculated with GFP-containing bacteria. Chi-square analysis revealed significant differences in detection between respective fetal tissues from inoculated and non-inoculated pregnancies (regardless of whether we analyzed 14 fetuses or 7 pregnancies). The statistical analysis therefore confirms that our detection of GFP could not be accounted for by false positive generation of PCR product.

**Table 3:** PCR amplification of plasmid DNA encoding Green Fluorescent Protein (GFP). Data are represented as number of fetuses in which there was positive detection and total number of fetuses tested for each tissue in inoculated and noninoculated pregnancies. Differences in rates of detection in inoculated versus noninoculated pregnancies were tested using Chi-Square analysis.

Green Fluorescent Protein (GFP)			
	Inoculated	Non-inoculated	Significance
**placentome**	13/14 (7/7 pregnancies)	0/7 (0/7 pregnancies)	*p* = 2.5 x 10^−4^
**spleen**	14/14 (7/7 pregnancies)	0/8 (0/7 pregnancies)	*p* = 2.3 x 10^−5^
**liver**	14/14 (7/7 pregnancies)	0/14 (0/7 pregnancies)	*p* = 8.9 x 10^−7^
**cortex**	14/14 (7/7 pregnancies)	0/7 (0/7 pregnancies)	*p* = 3.3 x 10^−4^

Our methodology for detection of RFP (Table 4) and FRFP (Table 5) appeared to be somewhat less sensitive (perhaps because of fragmentation of the RFP and FRFP plasmids in fetal tissues). Nevertheless, FRFP was statistically significantly detected in placentome (11/14 fetuses; 7/7 pregnancies), spleen (7/14 fetuses; 6/7 pregnancies), cerebral cortex (12/14 fetuses; 7/7 pregnancies), and liver (9/14 fetuses; 7/7 pregnancies). Detection of RFP was least sensitive: RFP was statistically significantly detected in placentome (10/14 fetuses; 6/7 pregnancies), spleen (5/14 fetuses; 3/7 pregnancies), cerebral cortex (6/14 fetuses; 4/7 pregnancies), but not in liver (1/14 fetuses; 1/7 pregnancies). In all cases, the negative controls (fetal tissues from non-inoculated pregnancies) did not amplify FRFP or RFP (Tables 4 and 5).

**Table 4:** PCR amplification of plasmid DNA encoding Red Fluorescent Protein (RFP). Data representation and statistical analysis are as described in Table 3.

Red Fluorescent Protein (RFP)			
	Inoculated	Non-inoculated	Significance
**placentome**	8/14 (6/7 pregnancies)	0/11 (0/11 pregnancies)	*p* = 9.1 x 10^−3^
**spleen**	5/14 (3/7 pregnancies)	0/14 (0/14 pregnancies)	*p* = 0.048
**liver**	1/14 (1/7 pregnancies)	0/14 (0/14 pregnancies)	*p* = NS
**cortex**	6/14 (4/7 pregnancies)	0/14 (0/14 pregnancies)	*p* = 0.02

**Table 5:** PCR amplification of plasmid DNA encoding far Red Fluorescent Protein (FRFP). Data representation and data analysis are as described in Table 3.

Far-Red Fluorescent Protein (FRFP)			
	Inoculated	Non-inoculated	Significance
**placentome**	11/14 (7/7 pregnancies)	0/14 (0/14 pregnancies)	*p* = 1.1 x 10^−4^
**spleen**	7/14 (6/7 pregnancies)	0/14 (0/14 pregnancies)	*p* = 8.8 x 10^−3^
**liver**	9/14 (7/7 pregnancies)	0/14 (0/14 pregnancies)	*p* = 1.2 x 10^−3^
**cortex**	12/14 (7/7 pregnancies)	0/7 (0/7 pregnancies)	*p* = 1.1 x 10^−3^

### Detection of immunoreactive GFP protein using immunohistochemistry

**Fig 1:**
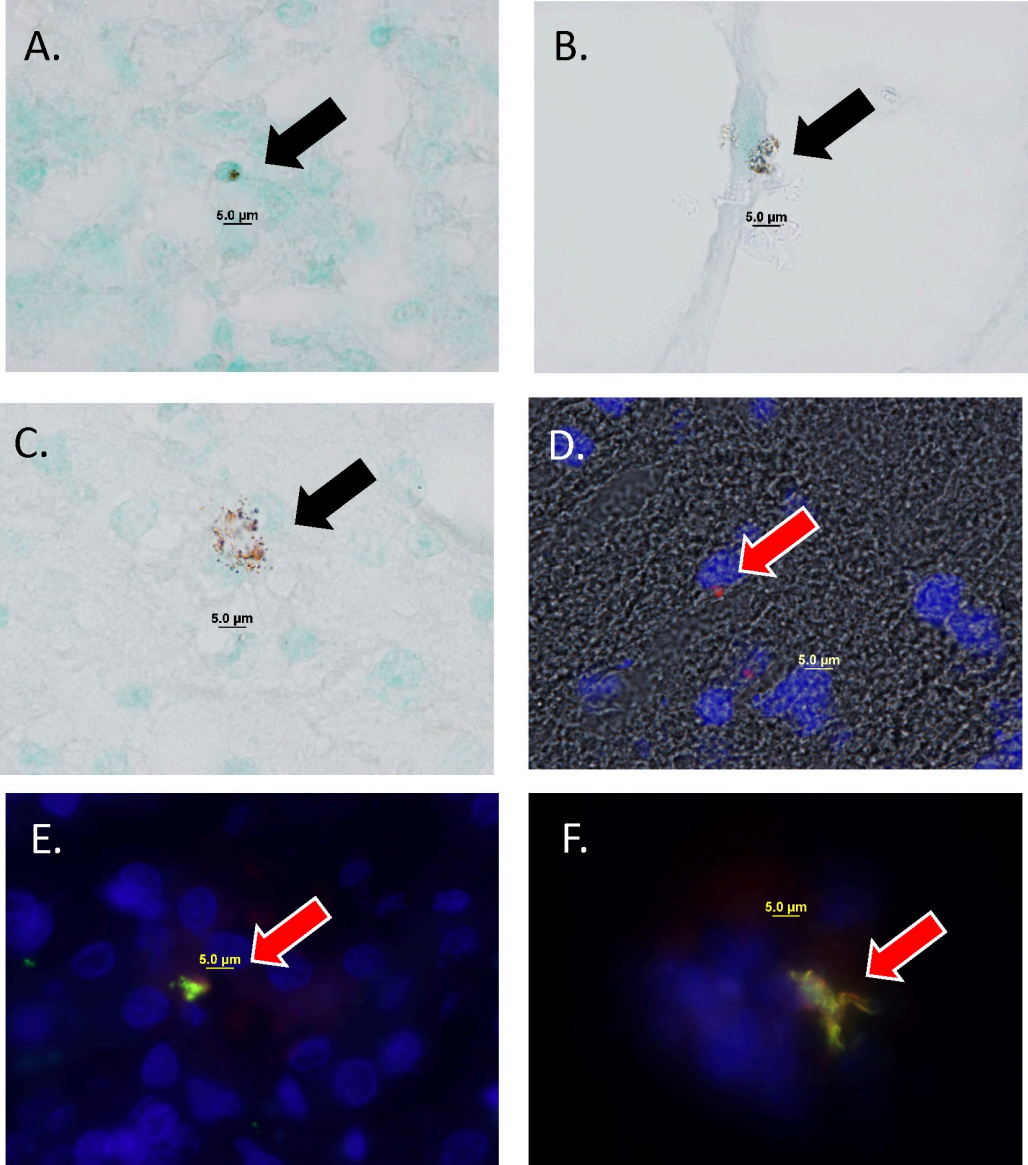
Immunohistochemistry (panels A-C) and immunofluorescence (panel D) images of green fluorescent protein-immunoreactive structures in fetal tissues 5–7 days post-inoculation of GFP-expressing *S*. *aureus* into the maternal bloodstream. Panel A, iGFP in fetal liver (fetus 6A). Panel B, iGFP in fetal cerebral cortex (fetus 6B). Panel C, iGFP in fetal cerebral cortex (fetus 20B). Panel D, merged image (brightfield, DAPI, red fluorescence) of immunofluorescence detection of GFP protein in fetal cerebral cortex (fetus 25B). In all panels images were recorded using oil immersion with a 100x microscope objective. Panels E and F, merged image (red anti-*S aureus*, green anti-GFP, blue DAPI) fetal liver (fetuses 6A and 6B, respectively).

Fetal cerebral cortex (n = 14 fetuses), fetal spleen (n = 14 fetuses), fetal liver (n = 14 fetuses), and placentome (n = 14 fetuses) were analyzed histologically for immunoreactive GFP protein. In all cases, we detected immunoreactive GFP protein in what appeared to be single or grouped bacterial cells with morphology similar to *S*. *aureus* as seen with light microscopy (round shape, 0.5–1 micron diameter). We detected intracellular GFP-immunoreactive structures with the correct shape (round) and size (0.5–1 micron diameter) to match the shape and size of *S*. *aureus*. These were noted in both the fetal liver (Fig 1A), the perivascular region of the fetal cerebral cortex (Fig 1B) and within the parenchyma of the cerebral cortex (Fig 1C). GFP labelling was found by immunofluorescence (Fig 1D, red color), as well as by immunohistochemical methods. Double labeling of iGFP and anti-*S aureus* confirms overlap of staining in fetal tissues (Fig 1).

### Detection of GFP protein using ELISA

**Fig 2:**
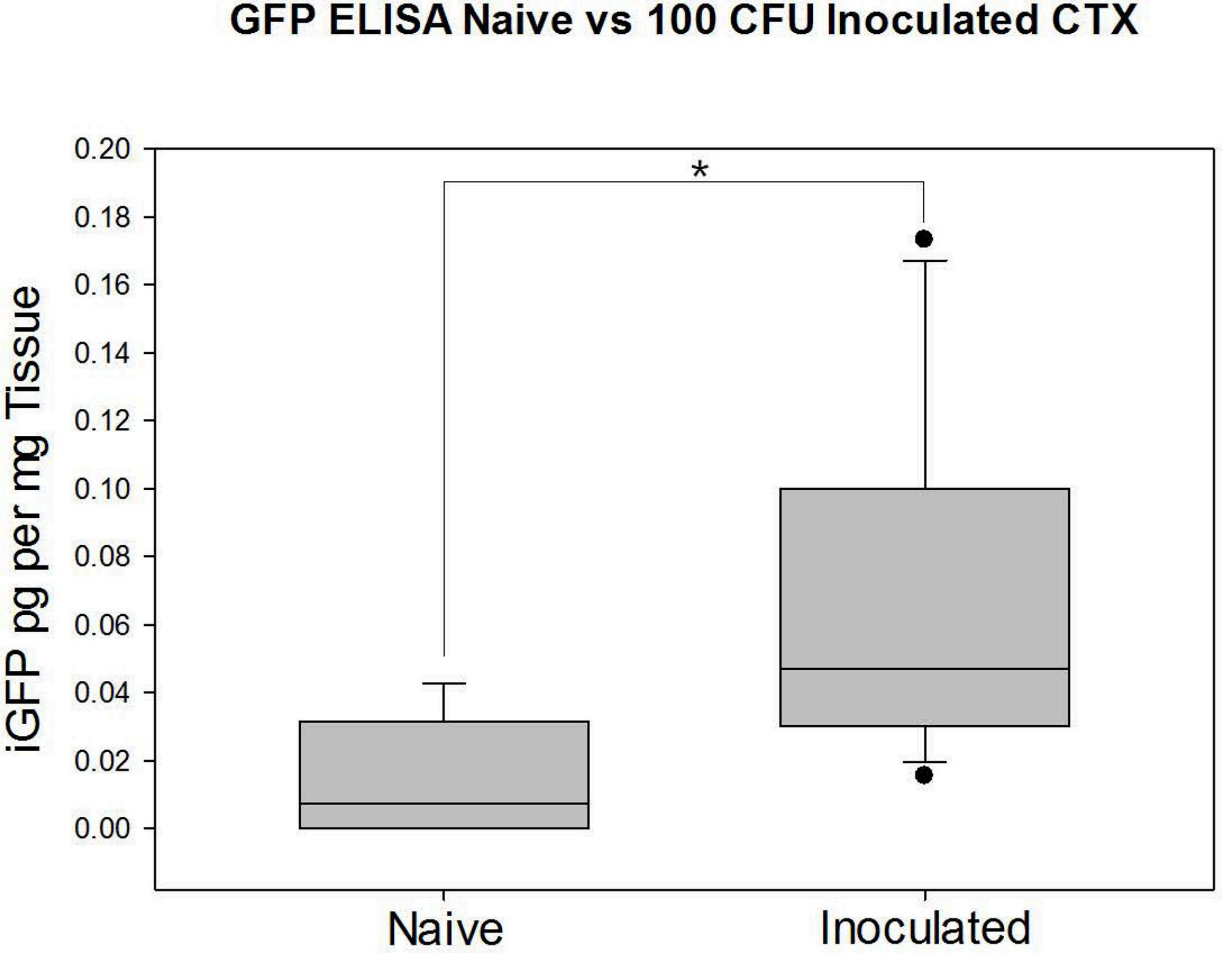
Immunoreactive GFP (iGFP) as measured by ELISA in cerebral cortex of fetuses from naïve (n = 6) and 100 cfu-inoculated (n = 14) pregnancies. Measured values of iGFP are significantly different between the two groups (p = 0.005).

Samples of fetal cerebral cortex (n = 14 fetuses inoculated fetuses and n = 6 naïve fetuses) were analyzed for immunoreactive GFP using a commercially-available ELISA method. Because extracted protein from tissue can include pigments that interfere with the colorometric endpoint in an ELISA and because of possible cross-reactivity of the anti-GFP antibody to other proteins, we analyzed tissue from fetuses from both naïve (not exposed to GFP) and GFP-*S*. *aureus*-inoculated pregnancies. The GFP immunoreactivity (iGFP) in the inoculated fetuses was significantly greater in the fetuses from inoculated pregnancies compared to the fetuses from naïve pregnancies (median iGFP 0.0471 pg/mg tissue vs. 0.0072 pg/mg, respectively). As shown in Fig 2, the data were not normally distributed, but comparison of groups by Mann-Whitney U test [14] revealed significant difference between groups (p = 0.005). Recognizing that twins in one uterus are not independent of each other, we also tested for significant differences between inoculated and naïve pregnancies when considering only one twin (twin A or twin B, names assigned arbitrarily at the time of sacrifice). When analyzed in this way, the distribution of the data allowed use of Student’s t-test which resulted in rejection of the null hypothesis for each comparison (p = 0.02 and p = 0.03 for naïve vs. twin A and twin B, respectively).

### Detection of live bacteria

We were unable to detect live GFP-containing bacteria by culture in fetal tissues collected from inoculated animals. It is noteworthy, however, that we were able to successfully culture bacteria from fetuses whose mothers had been inoculated with 10^4^, 10^6^, or 10^8^ cfu; some of those ewes also had symptoms of infection, including elevated maternal body temperature (data not reported in this report).

## Discussion

We have confirmed our hypothesis that *S*. *aureus* introduced into the maternal bloodstream crosses the maternal/fetal barrier and enters into the fetus. While we were able to demonstrate the appearance of GFP DNA and protein in the fetus, we were unable to demonstrate that the GFP-containing bacteria in the fetus were alive. Our inability to find live GFP-expressing bacteria in the fetus might be the result of the time interval allowed between inoculation and tissue collection (6 days). The design of the experiment does not allow us to determine whether the bacteria were killed upon transit from mother to fetus or whether the bacteria entered the fetus alive, then were killed by the fetal immune system. Consistent with the absence of detectable live bacteria is the appearance of the GFP protein in the tissue, which appeared as aggregated clumps rather than with morphology consistent with intact live bacteria. The appearance of bacteria in aggregated clumps as well as their low abundance in the fetal tissues may explain the discordance amongst labels (GFP, RFP, FRFP) in detection of plasmid DNA in fetal livers. The DNA extraction protocol uses approximately 100-150mg of tissue and RFP bacteria may have been absent in those specific tissue pieces used for extraction.

While it is clear that, at the minimum, we can find the remnants of our fluorescent protein-containing bacteria in the fetus–proof of concept that the fetus is exposed to bacteria in the environment–the present experiment did not prove that the bacteria are transferred to the fetus as live organisms. The small numbers of bacteria that we used to inoculate the sheep (100 cfu of each of 3 fluorescent protein-containing bacteria) and the abundance of plasmid DNA and protein in the fetus 5–7 days later is consistent with expansion of the inoculum at some point prior to the collection of tissue, although we were unable to demonstrate any expansion in the maternal blood in our daily blood samples. Our inability to find live FP-containing bacteria in the fetus strongly suggests that the immune system of either the mother or the fetus killed the FP-containing bacteria and, 5–7 days post inoculation, was in the process of clearing them from the fetus. A limitation of our experimental design is that we did not look for viable bacteria sooner than 5–7 days post inoculation. It is possible that the inoculated bacteria are expanded in the mother and that a portion of the expanded population is transferred to the fetus live. It is also possible that the bacteria expand and are killed in the mother and that bacterial cell antigens and fragments are transferred to the fetus [15]. If so, the fetus responds to the bacterial invasion with a specific immune response, as shown in the mouse model [15]. This immune response would ultimately inform the development of the fetal immune system and ready the fetus for life outside the uterus [15].

Our previous experiments [4] prove that after hypoxia–a stimulus that disturbs maternal and fetal physiology and homeostasis–there was a transfer of live bacteria from placenta to fetus. Transcriptomics analysis revealed that accompanying the influx of bacteria is a transcriptomics signature that is consistent with increased immune and inflammation responses [5]. In the absence of hypoxia, one might ask, is there evidence of exposure to bacteria? Elegant experiments in the mouse model clearly demonstrate that bacteria introduced into the maternal mouth inform the development of the innate immune system of the offspring [15]. Maternal-fetal transfer in those experiments was largely dependent on maternal IgG, which is known to be transferred across the placenta in both mouse [16] and human [17], although IgG transfer is not thought to occur in sheep [18]. Interestingly, though, recent transcriptomics modeling of ontogenetic patterns of gene expression in the fetal brain demonstrate that the major pattern of increasing gene expression in the fetal brain is the development of hematopoietic immune cells [19]. This was evident in fetuses with no surgical manipulation. Indeed, these patterns of gene expression are consistent with progressive exposure of the fetus to pathogen-associated molecular patterns that might well include bacterial cell components.

An unanswered question, related to a limitation of our study, is whether this transfer of bacteria occurs in other species. We used the sheep model because of the size and experimental manipulability of the fetus, and because of its relation to our previous experiments investigating responses to hypoxia [5, 6] and ontogenetic patterns of gene expression [19]. Reports of bacterial DNA in the human placenta and neonatal meconium based on DNA sequencing technology alone have been challenged on the basis of potential contamination artifact results. Nevertheless, several studies have reported the presence of bacterial DNA [7] and intact bacterial cells [8, 9] in the human placenta. Recent studies by several investigators have demonstrated the presence of bacteria in the meconium of newborn infants [20–23]. While the present sheep experiments are not possible to perform in the human, we are intrigued by the possibility that fetal exposure to bacterial antigens cleared from the maternal circulation might not be limited to the sheep.

The possibility of exposure of the fetus to bacteria is a controversial topic [10, 24]. The placenta is thought to be an effective barrier for microbes [1, 25, 26]. For most of the last century researchers believed that the fetus is sterile until parturition [27] and that bacteria colonizes the newborn during parturition or quickly after through contact with air, vagina, or breast [10, 28, 29]. Previous studies have highlighted the innate defense mechanisms of the placenta, and therefore the natural placental barrier that protects the fetus from microbial infection [25, 30]. The results of the present experiment does not contradict the concept of immune protection of the fetus. Rather, the present results provide proof of concept that the fetus is exposed to the pathogen-associated molecular patterns of the bacteria in an inoculum containing small numbers of bacteria–too few to cause a physiological fever or behavioral response in the pregnant ewe. A core question that remains to be investigated is whether these bacteria are killed by the maternal immune system prior to or during transplacental transfer.

## Supporting information

S1 Table ELISA results. (XLSX)

S2 Table PCR plasmid results. (XLSX)

S1 Fig Gel images. (DOCX)
