# Supplementary material for: Proof of principle: Physiological transfer of small numbers of bacteria from mother to fetus in late-gestation pregnant sheep
Source: PLoS One. 2019 Jun 6;14(6):e0217211. doi: 10.1371/journal.pone.0217211 (PMC6553719; doi:10.1371/journal.pone.0217211)
Supplement: S1 Fig — (DOCX) [file pone.0217211.s003.docx]

**Key:**

COTY = Cotyledon/Placentome

SPL = Spleen

LIV = Liver

CTX = Brain Cortex

GFP Plasmid = Positive control

Water = Negative control


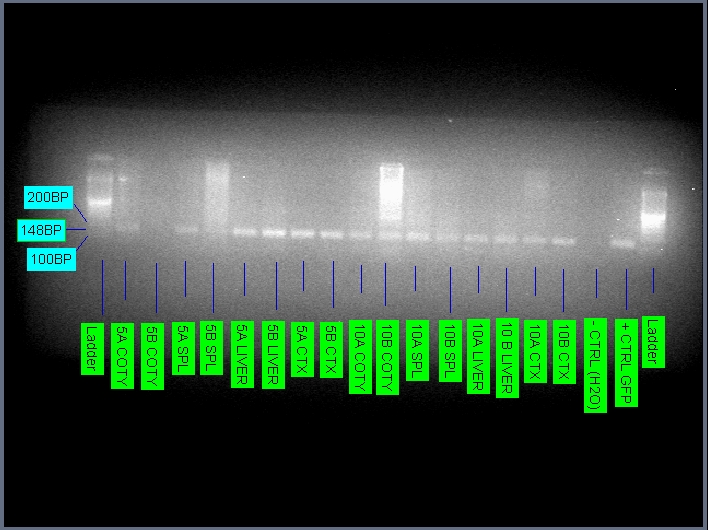


GFP bands from left to right: 100bp ladder, 5A COTY, 5B COTY, 5A SPL, 5B SPL, 5A LIV, 5B LIV, 5A CTX, 5B CTX, 10A COTY, 10B COTY, 10A SPL, 10B SPL, 10A LIV, 10B LIV, 10A CTX, 10B CTX, Water, GFP Plasmid, 100 bp ladder


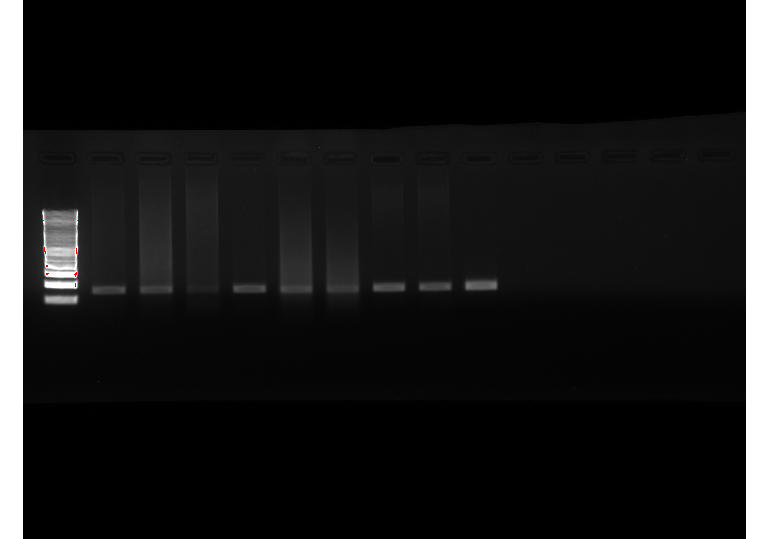


GFP Bands from left to right: 100bp ladder, 2A COTY, 2B COTY, 2A SPL, 2B SPL, 2A LIV, 2B LIV, 2A CTX, 2B CTX, GFP Plasmid


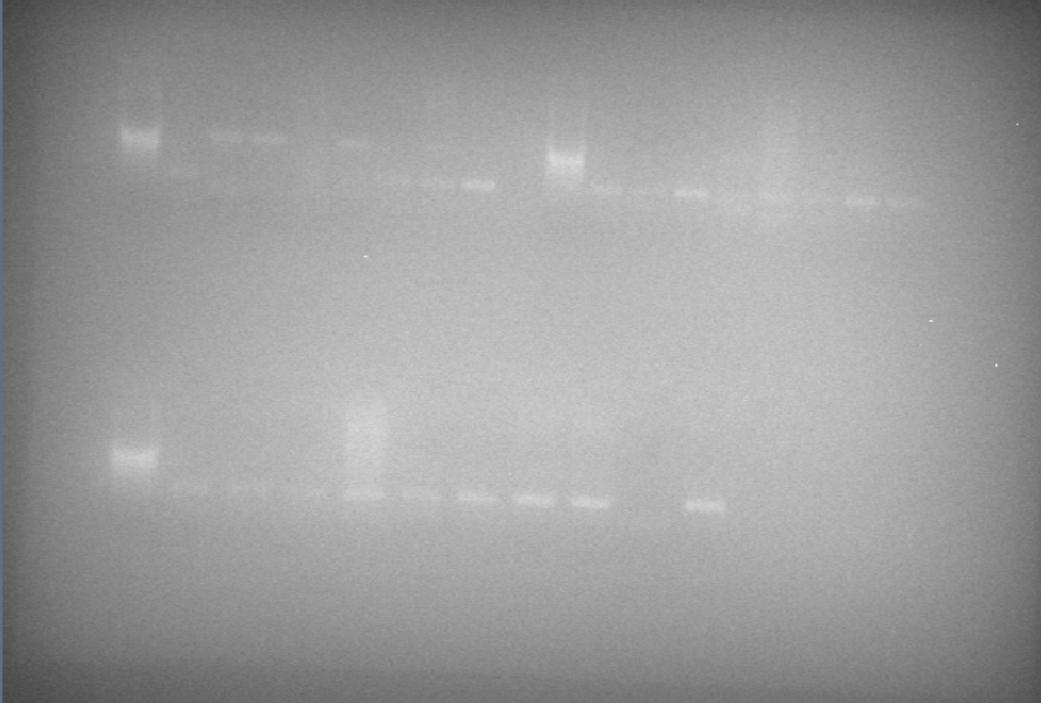


GFP Bands. Top Gel from left to right: 100bp ladder, 25A CTX, 25B CTX, 25A SPL, 25B SPL, 25A LIV, 25B LIV, 25A CTX, 25B CTX, Empty, 100bp ladder, 20A CTX, 20B CTX, 20A SPL, 20B SPL, 20A LIV, 20B LIV, 20A CTX, 20B CTX

Bottom Gel from left to right: 100bp ladder, 32A COTY, 32B COTY, 32A SPL, 32B SPL, 32A LIV, 32B LIV, 32A CTX, 32B CTX, Water, GFP Plasmid(~148bp)


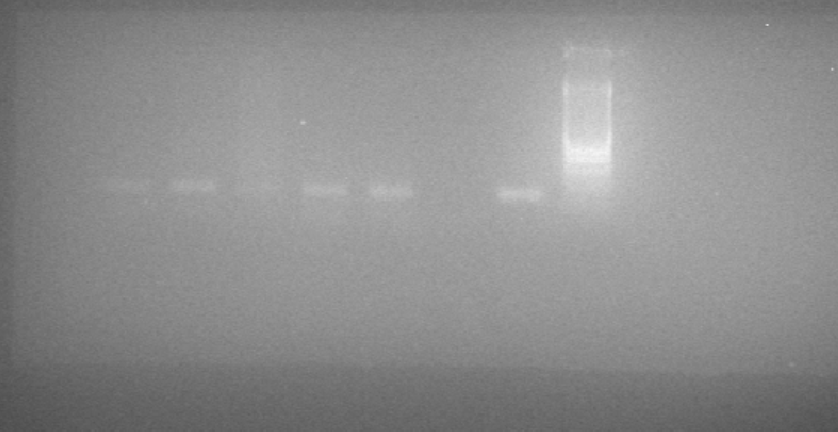


Confirmation gel for previous bands that were unclear in picture.

GFP bands from left to right: 25B COTY, 25A SPL, 52B SPL, 25A LIV, 20B SPL, Water, GFP plasmid(~148bp), 100bp ladder.


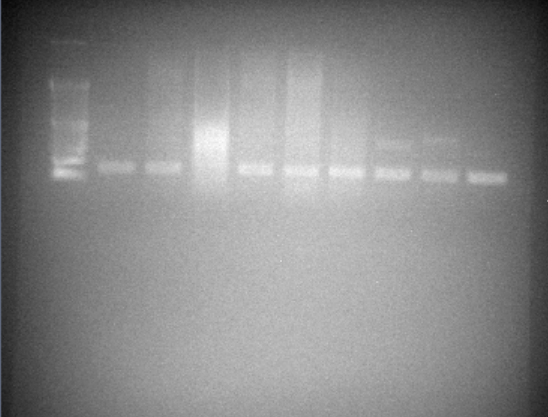


GFP Bands from left to right: 100bp ladder, 6A COTY, 6B COTY, 6A SPL, 6B SPL, 6A LIV, 6B LIV, 6A CTX, 6B CTX, GFP Plasmid (~148bp)


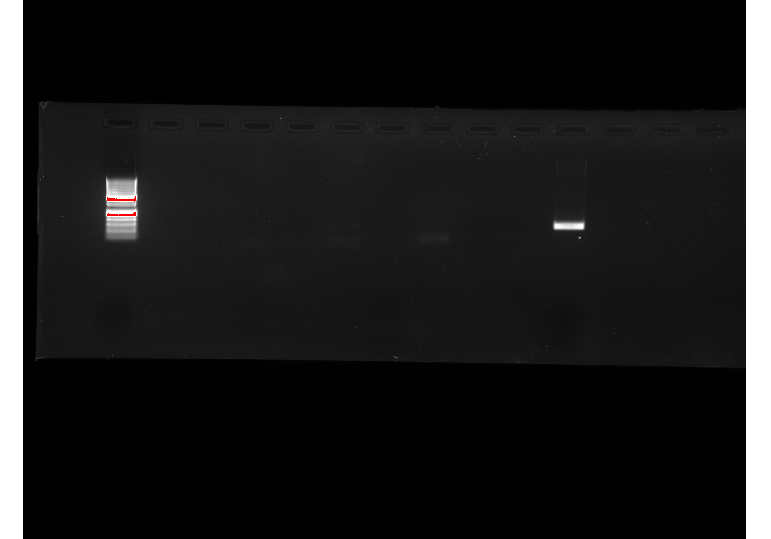


GFP Negative controls spleen. From left to right: 50bp ladder, 5580A SPL, 5562A SPL, 5562B SPL, 5562C SPL, 5320A SPL, 1481A SPL, 1562A SPL, 5563B SPL, Water, GFP Plasmid


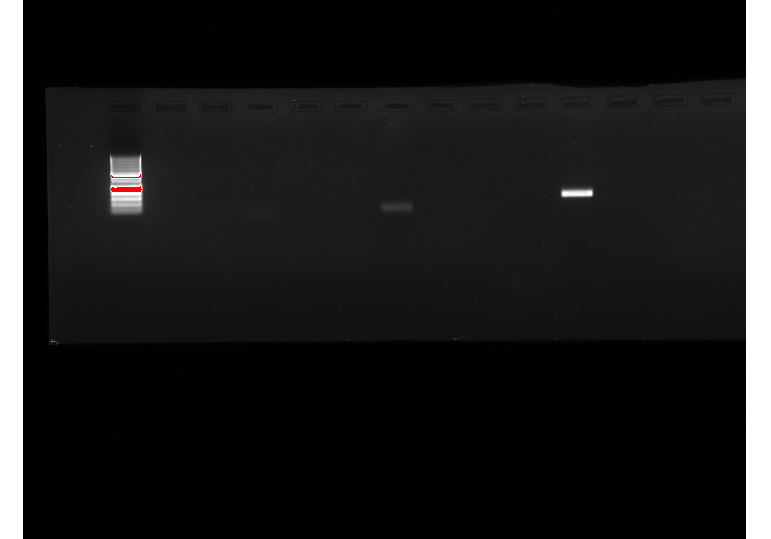


GFP Negative controls placentome. From left to right: 50bp ladder, 5580A COTY, 5580B COTY, 5320A COTY, 1481A COTY, 1562A COTY, 5579B COTY, 5579A COTY, Water, Empty, GFP Plasmid


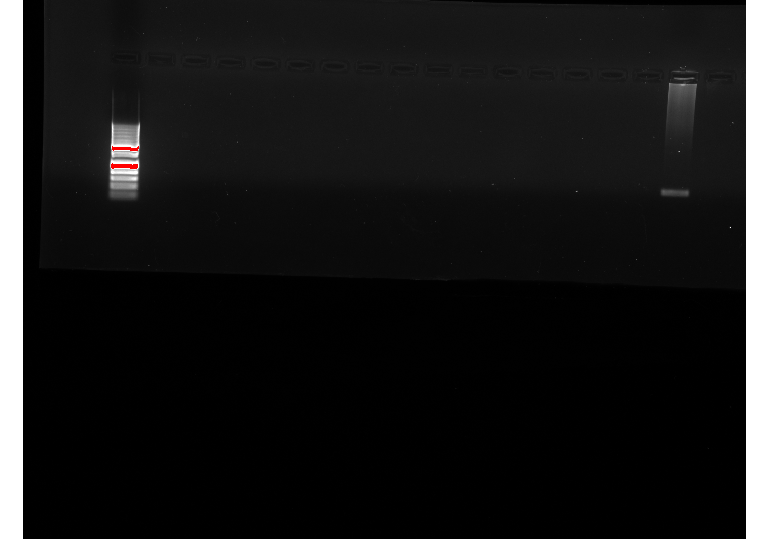


GFP Band Negative Liver controls from left to right: 50bp ladder, R5A, Y861, Y850, 4849A, R48, W6, W74, 891A, Y97A, 891B, 500A, FetB, Y868, 500B, Water, GFP Plasmid


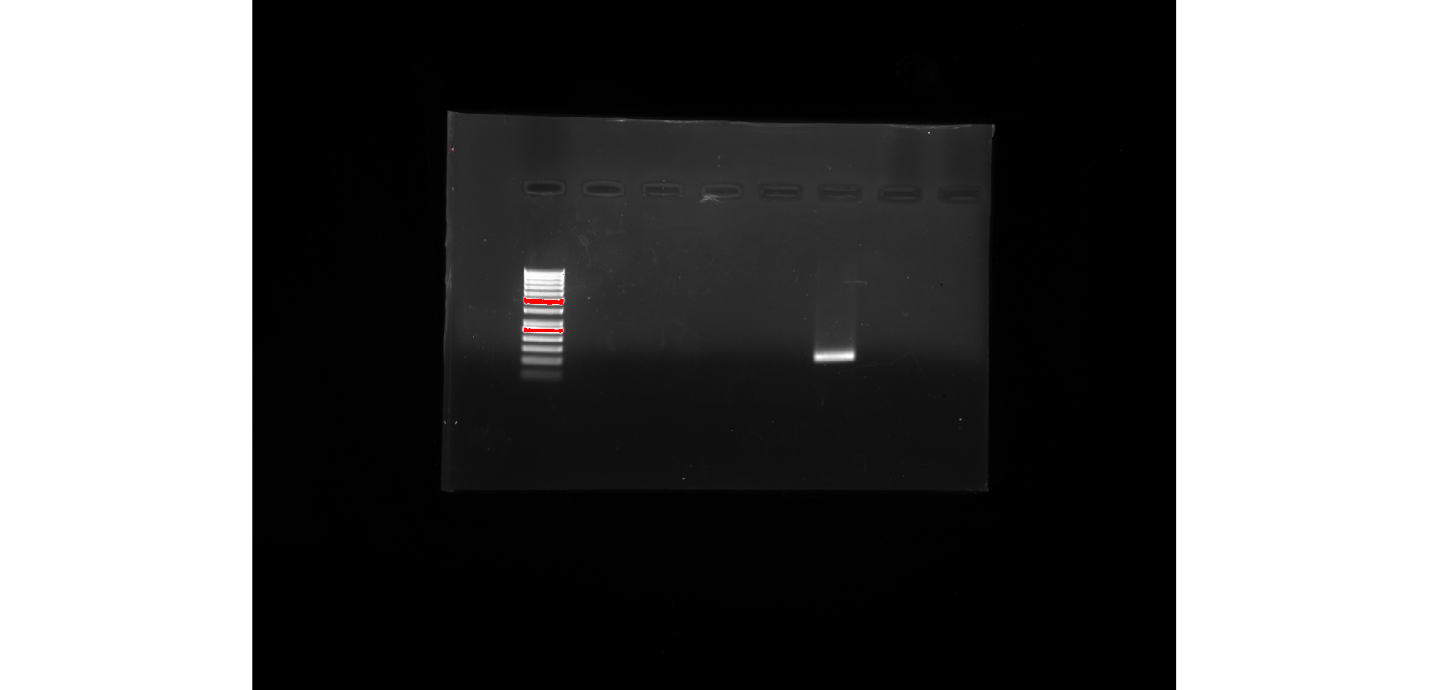


GFP Band Negative controls from left to right: 50bp ladder, 5563 SPL, 5579 COTY, W74 LIV, Water, GFP Plasmid


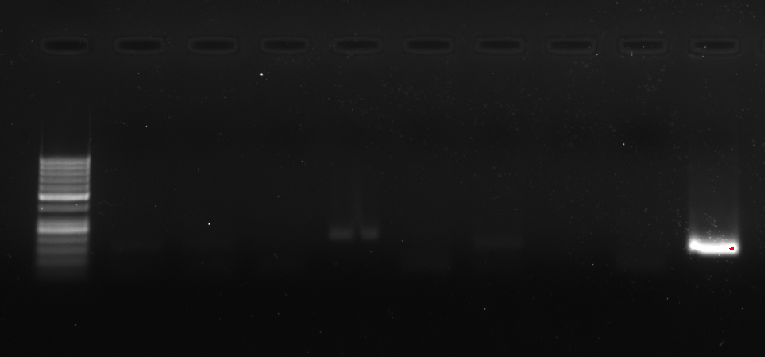


GFP Band Negative control from left to right: 100bp ladder, 5580A CTX, 5580B CTX, 5562A CTX, 5562B CTX, 5320A CTX, 1481A CTX, 1562A CTX, Water, GFP Plasmid


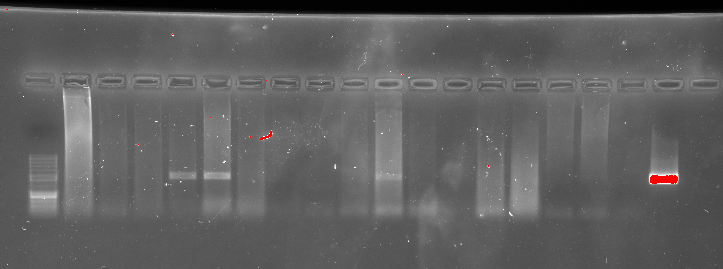


RFP Bands from left to right: 50bp ladder, 6A COTY, 6B COTY, 6A SPL, 6B SPL, 6A LIV, 6B LIV, 6A CTX, 6B CTX, 2A COTY, 2B COTY, 2A SPL, 2B SPL, 2A LIV, 2B LIV, 2A CTX, 2B CTX, Water, RFP Plasmid


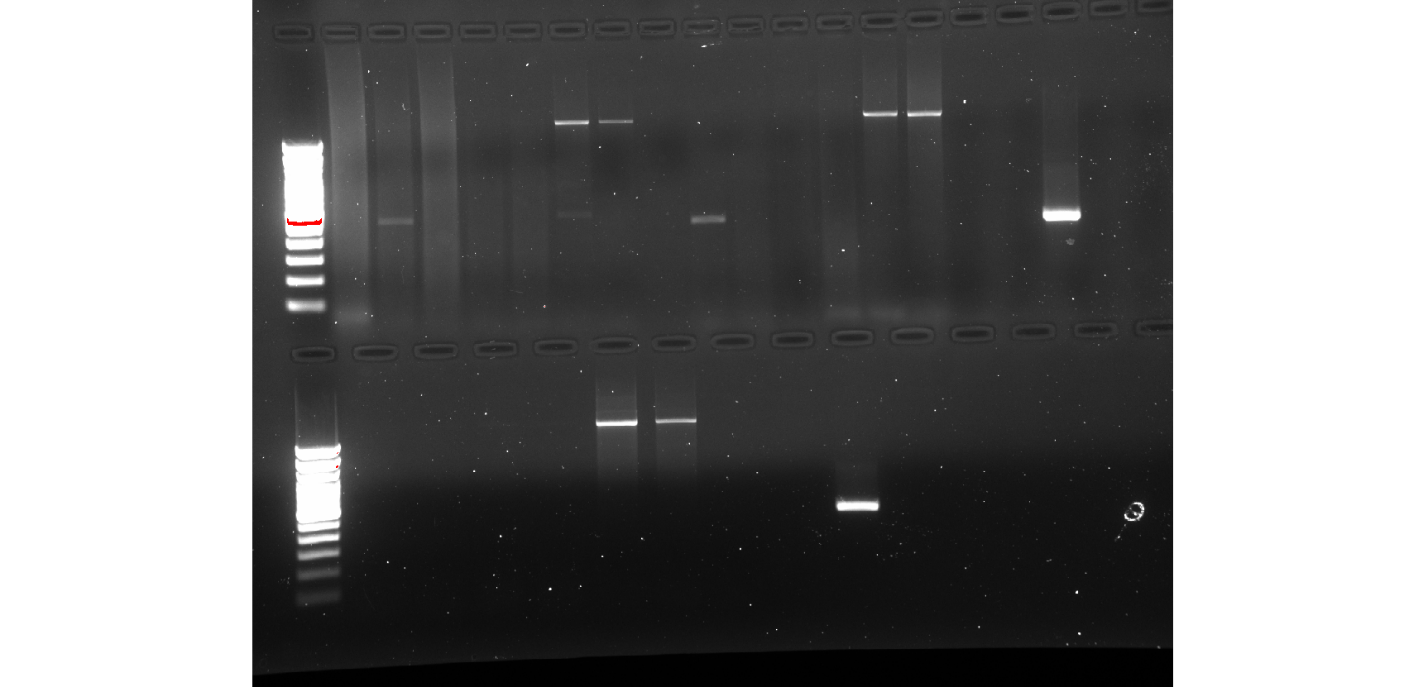


RFP bands on Upper gel from left to right: 100bp Ladder, 20A LIV, 20A SPL, 5B SPL, 5A LIV, 5B LIV, 5A CTX, 5B CTX, Empty, 25A LIV, 25B LIV, 25A SPL, 25B SPL, 25A CTX, 25B CTX, Water, RFP Plasmid.

Bottom gel from left to right: 100bp ladder, 32A LIV, 32B LIV, 32A SPL, 32B SPL, 32A CTX, 32B CTX, Water, Empty, RFP Plasmid.


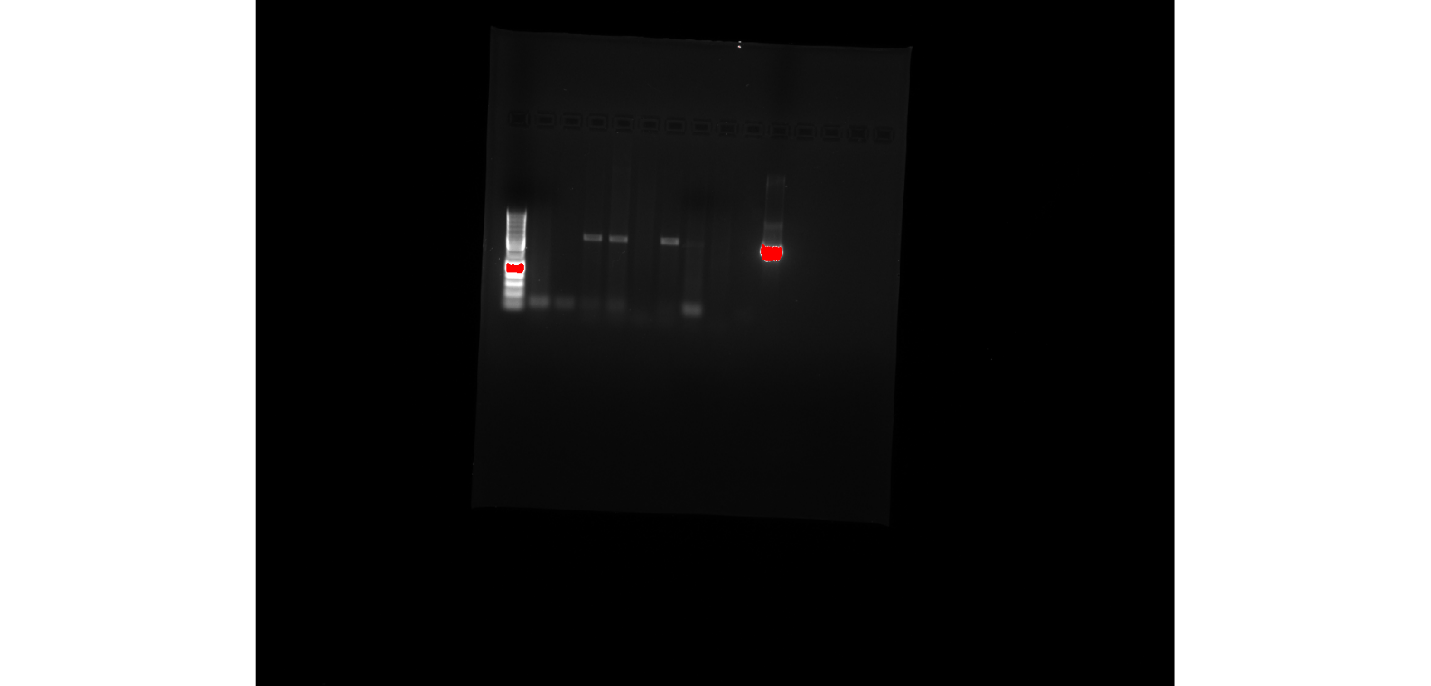


RFP bands from left to right: 50bp ladder, 25A COTY, 25B COTY, 32A COTY, 32B COTY, 5A COTY, 5B COTY, 5A SPL, 5B LIV, Water, GFP plasmid


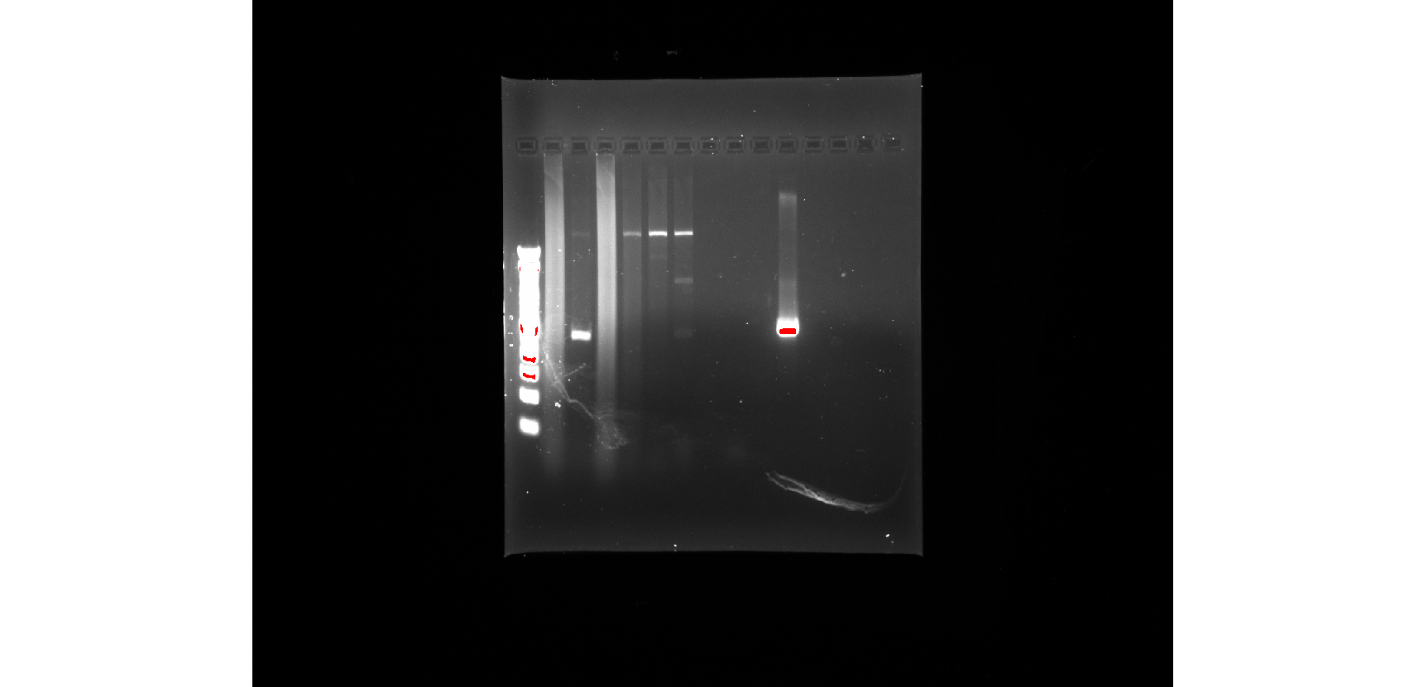


RFP bands from left to right: 100bp Ladder, 20A SPL, 20B SPL, 20A LIV, 20B LIV, 20A CTX, 20B CTX, 20A COTY, 20B COTY


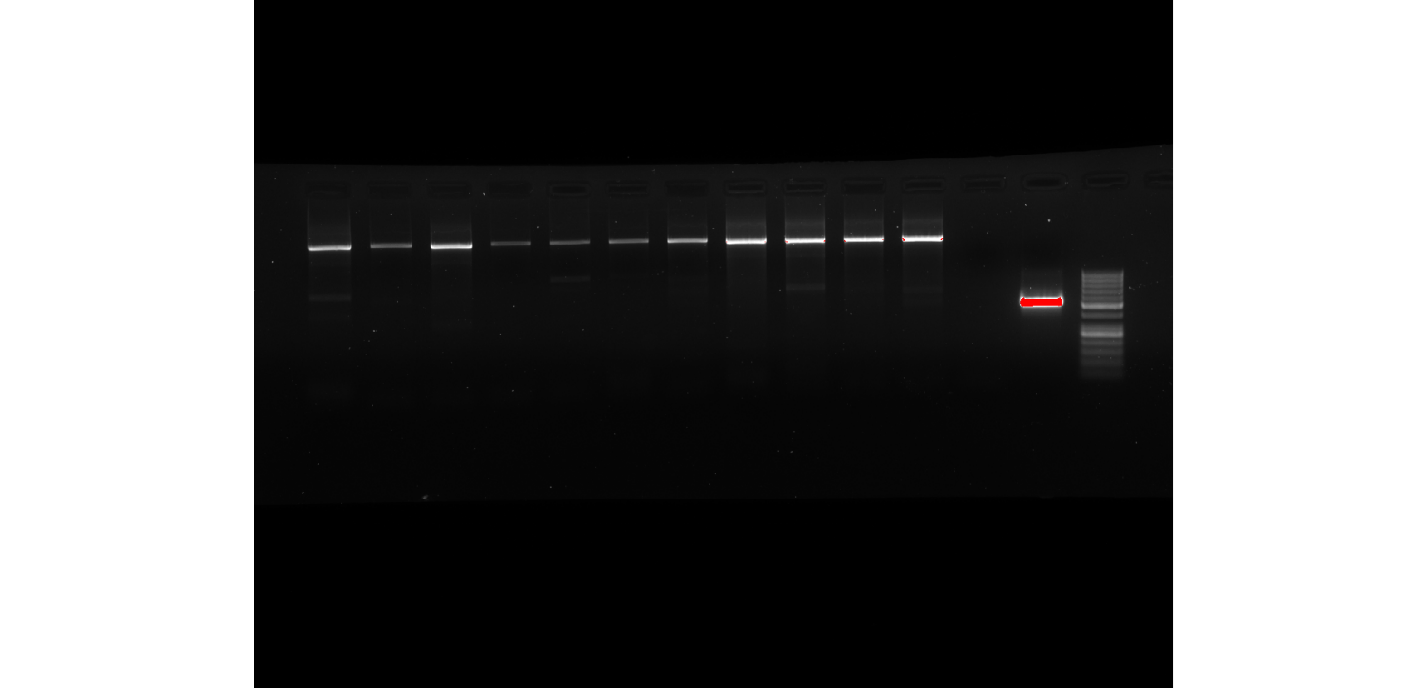


RFP negative control band from Left to right: 50bp Ladder, 1462 CTX, 1477 CTX, 1767A CTX, 1767B CTX, 1555A CTX, 1763 CTX, 1237B CTX, 1237B COTY, 1237A COTY, 1335A COTY, 5563B COTY, Water, RFP Plasmid, and Ladder


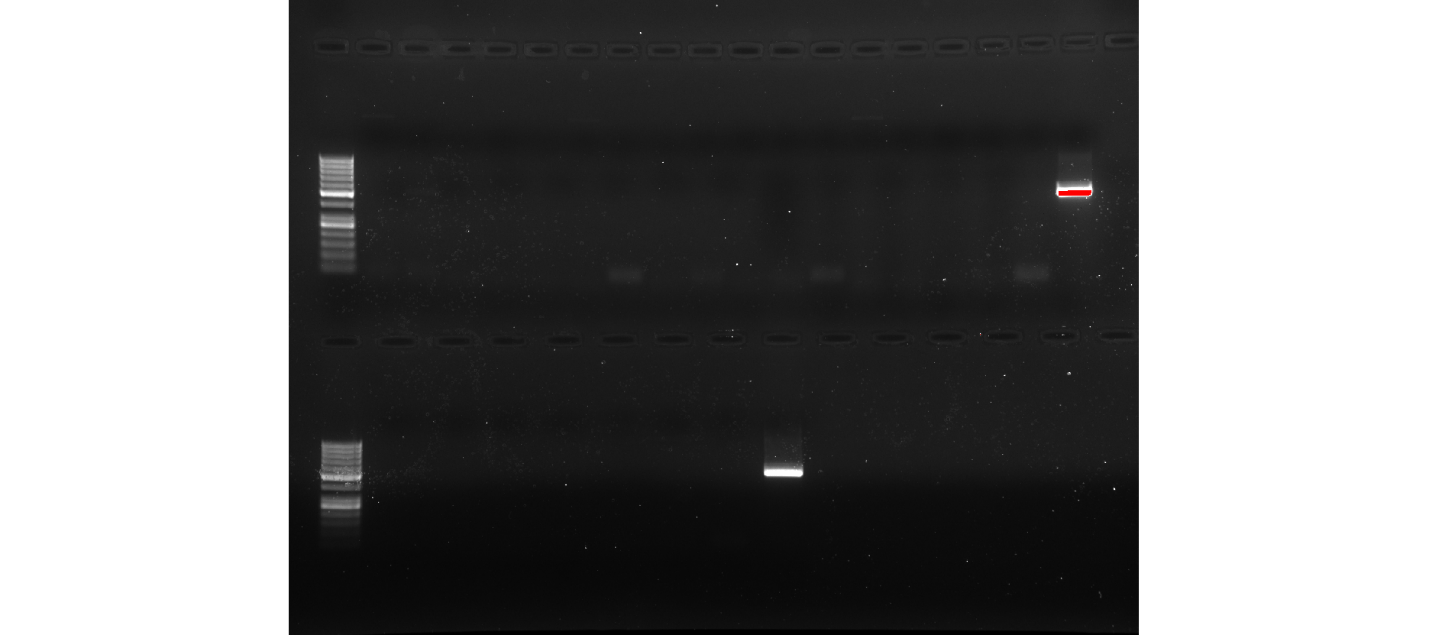


RFP Negative controls. Top gel from left to right: 50bp ladder, 5580A CTX, 5580B CTX, 5562A CTX, 5562B CTX, 5320A CX, 1481A CTX, 1562A CTX, 5580A SPL, 5562A SPL, 5562B SPL, 5562C SPL, 5320A SPL, 1481A SPL, 1562A SPL, 5563B SPL, 5580A COTY, 5580B COTY, RFP Plasmid

Bottom Gel from left to right: 50bp ladder, 5320A COTY, 1481A COTY, 1562A COTY, 5563B COTY, 5579A COTY, 5579B COTY, Water, RFP Plasmid


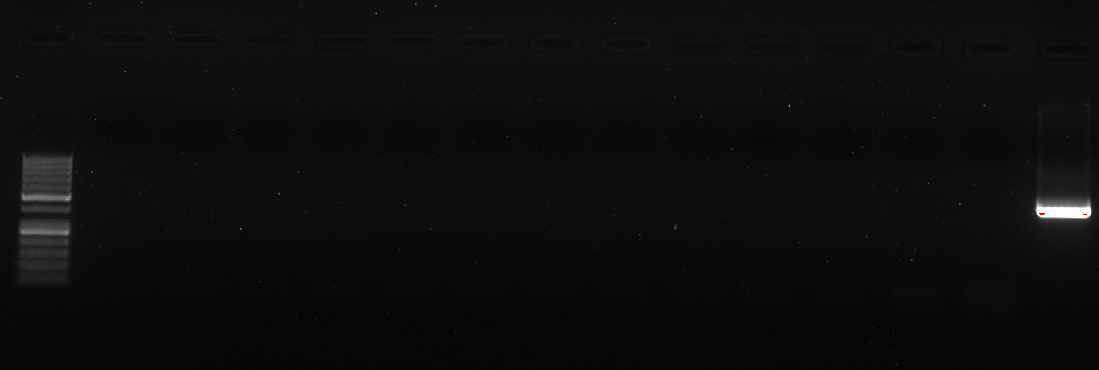

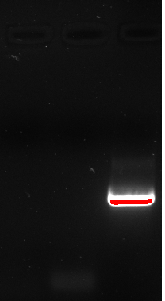


RFP Negative Liver Controls: Top picture form left to right: 50bp ladder, Y850, 4849A, R58A, W6, W74, 891A, Y97B, 891B, 500A, FetB, Y868, 500B, Water, RFP Plasmid(~513bp)

Bottom Picture from left to right: R5A, Y861, RFP Plasmid(~513bp)


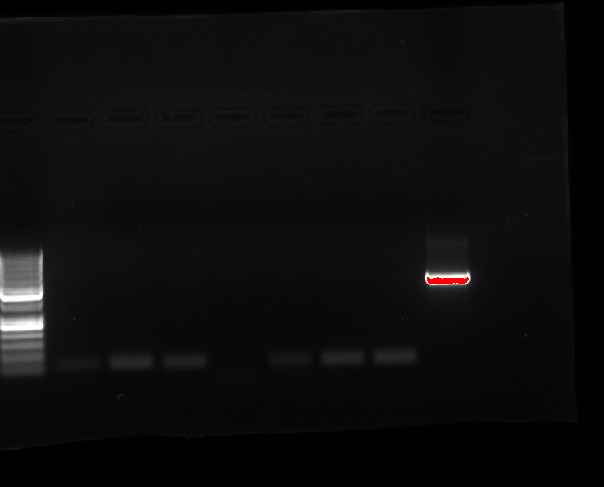


RFP Negative control spleen from left to right: 50bp ladder, R58, Y972, FetB, 500B, W71, Y960, Water, RFP Plasmid


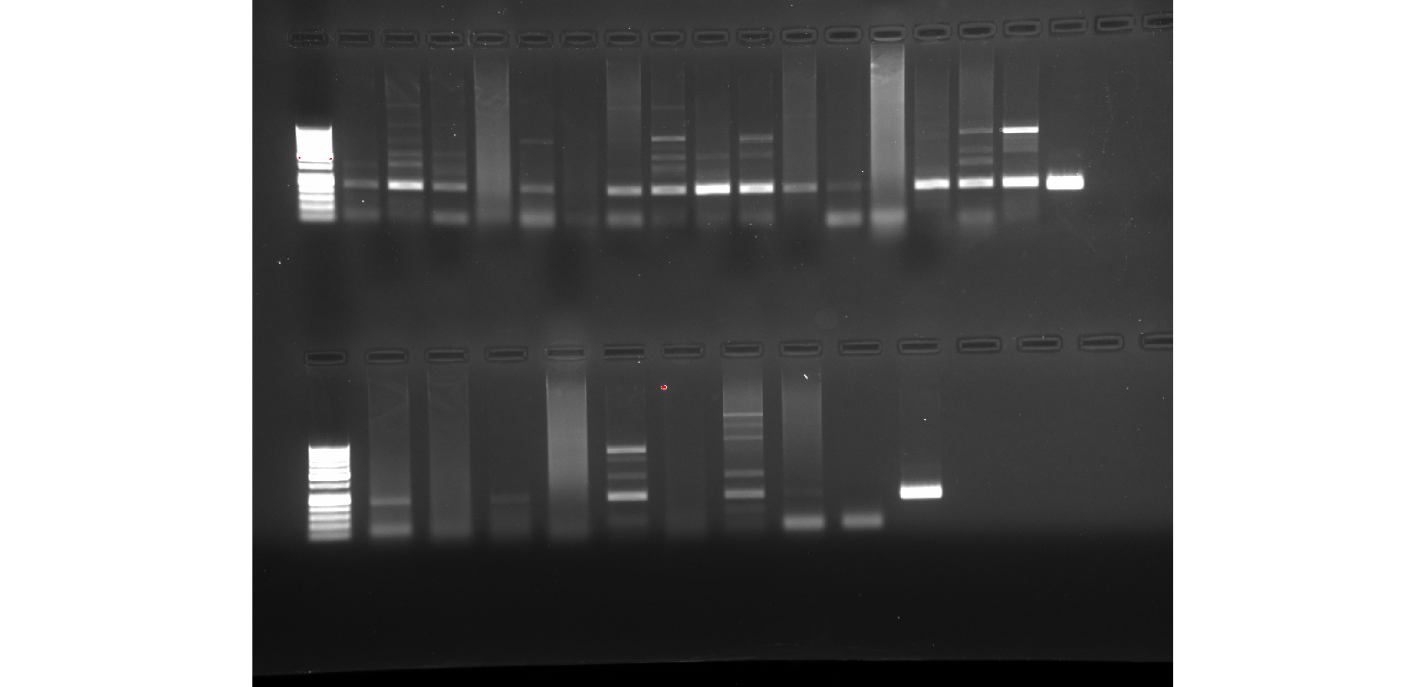


FP650 Bands Top gel from left to right: 50bp ladder, 25A COTY, 25B COTY, 25A SPL, 25B SPL, 25A LIV, 25B LIV, 25A CTX, 25B CTX, 20A COTY, 20B COTY, 20A SPL, 20B SPL, 20A LIV, 20B LIV, 20A CTX, 20B CTX, FP650 Plasmid.

Bottom Gel from left to right: 50bp ladder, 32A COTY, 32B COTY, 32A SPL, 32B SPL, 32A LIV, 32B LIV, 32A CTX, 32B CTX, Water, FP650 plasmid


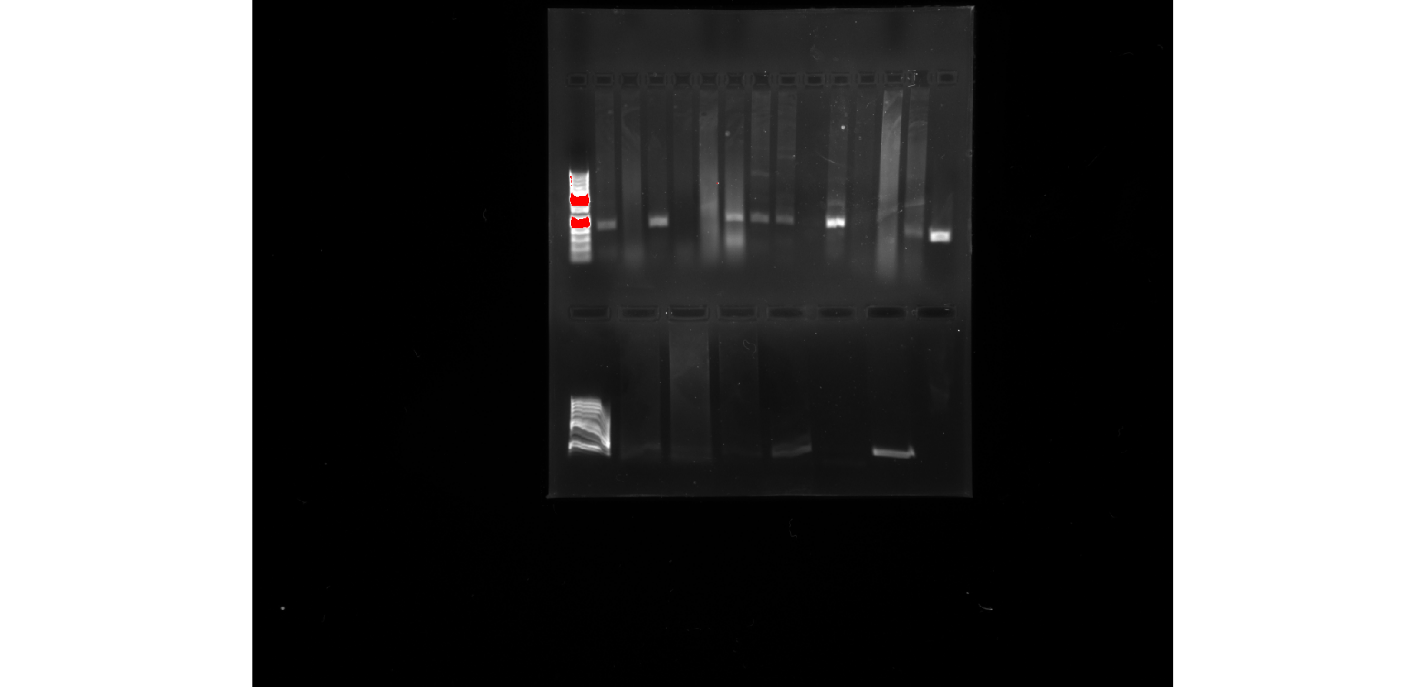


FP650 bands from left to right: 50bp ladder, 2A COTY, 2B COTY, 2A SPL, 2B SPL, 2B LIV, 2A LIV, 2A CTX, 2B CTX, Empty, 6A COTY, 6B COTY, 6A SPL, 6B SPL, FP650 Plasmid

Bottom Gel from left to right: 50bp ladder, 6A LIV, 6B LIV, 6A CTX, 6B CTX, Water, FP650 Plasmid


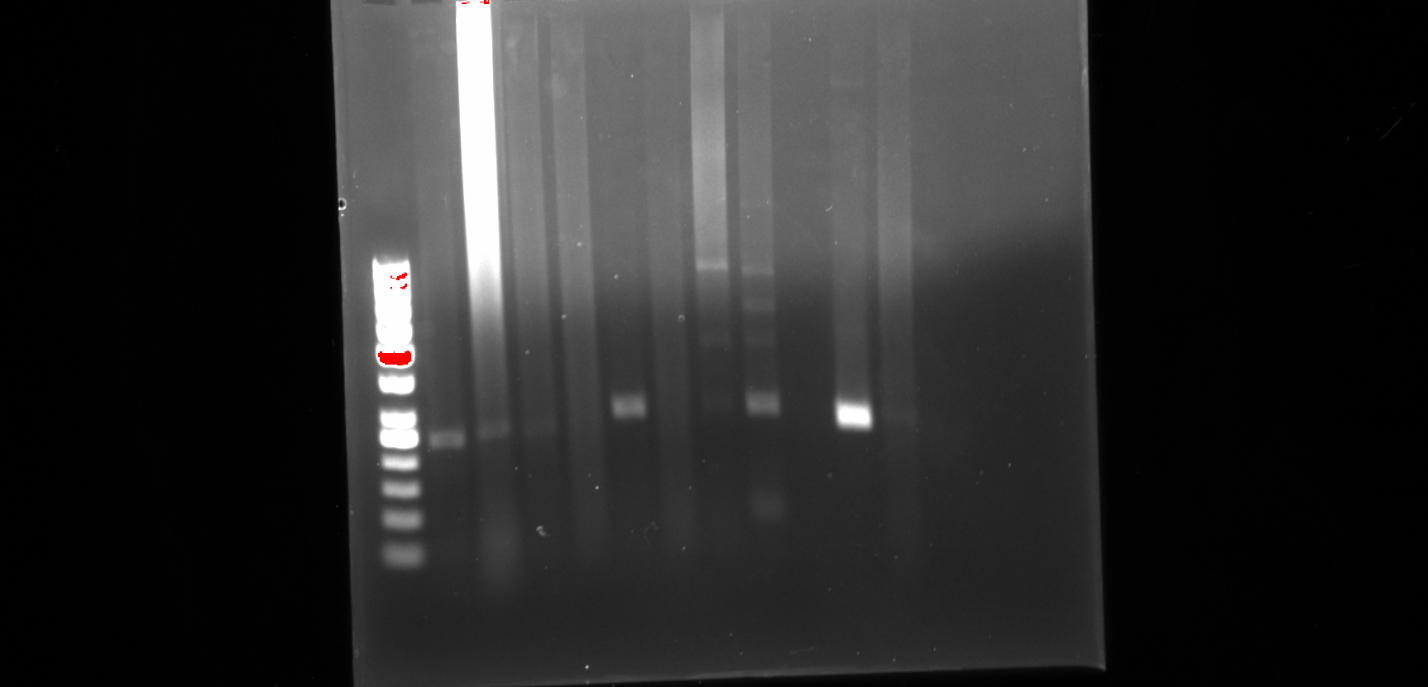


FP650 Bands from left to right: 50bp ladder, 10A COTY, 10B COTY, 10A SPL, 10B SPL, 10A LIV, 10B LIV, 10A CTX, 10B CTX, Water, FP650 Plasmid


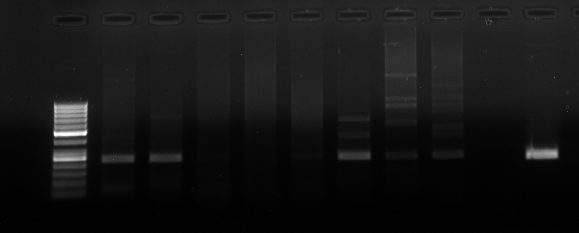


FP650 Bands from left to right: 50bp ladder, 5A COTY, 5B COTY, 5A SPL, 5B SPL, 5A LIV, 5B LIV, 5A CTX, 5B CTX, Water, FP650 Plasmid


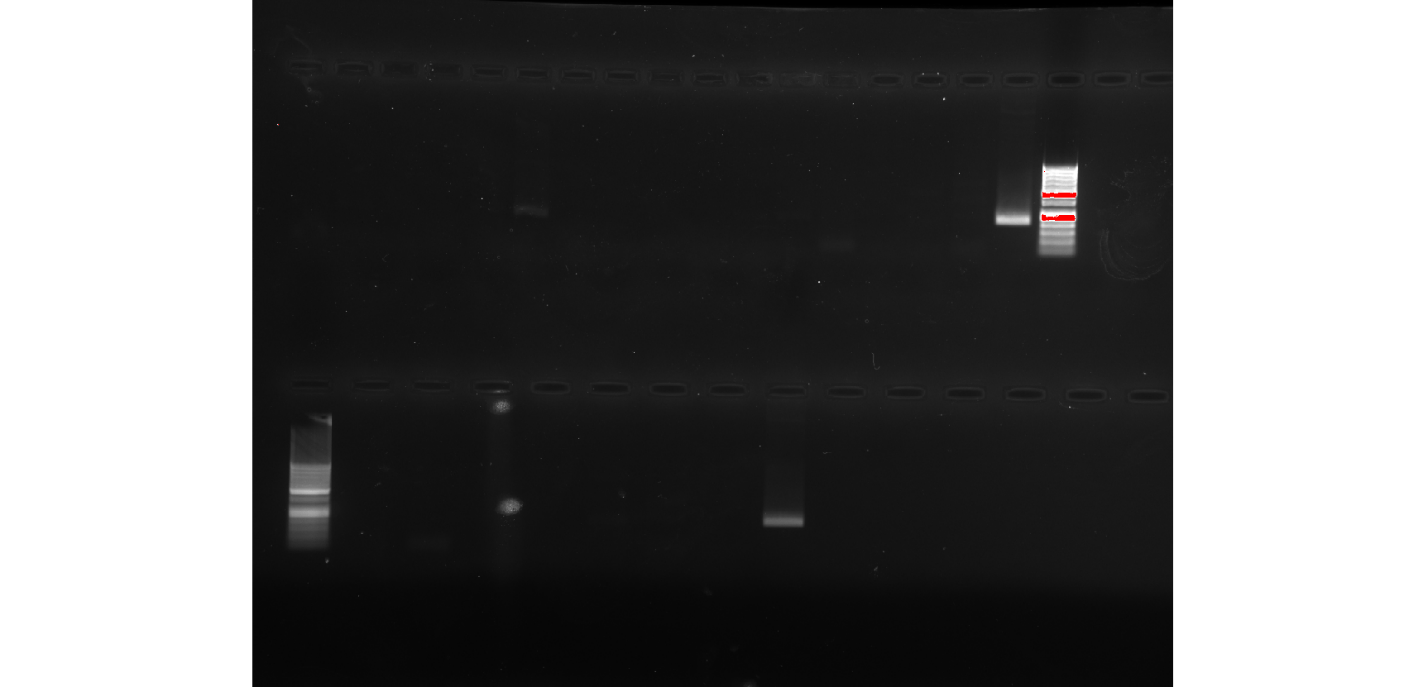


FP650 Negative controls Top gel from left to right: 5580A CTX, 5580A SPL, 5580A COTY, 5580B CTX, 5580B COTY, 5562A CTX, 5562A SPL, 5562B CTX, 5562B SPL, 5562C SPL, 5320A CTX, 5320A SPL, 5320A COTY, 1481A CTX, 1481A SPL, 1481A COTY, FP650 Plasmid, 50bp ladder.

Bottom gel from left to right: 50bp Ladder, 1562A CTX, 1562A SPL, 1562A COTY, 5563B SPL, 5579A COTY, 5579B COTY, Water, FP650 Plasmid.


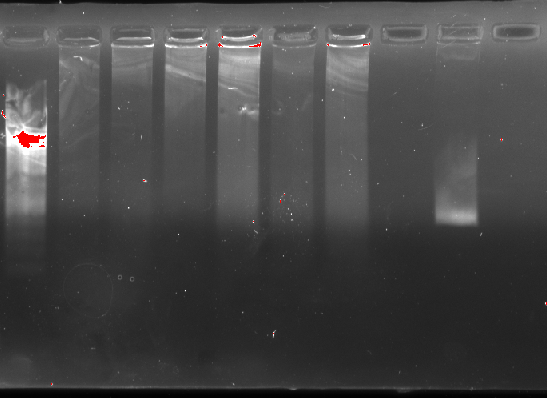


FP650 Negative control spleen from left to right: 50bp ladder, R58, Y972, FetB, 500B, W71, Y960, Water, FP650 Plasmid


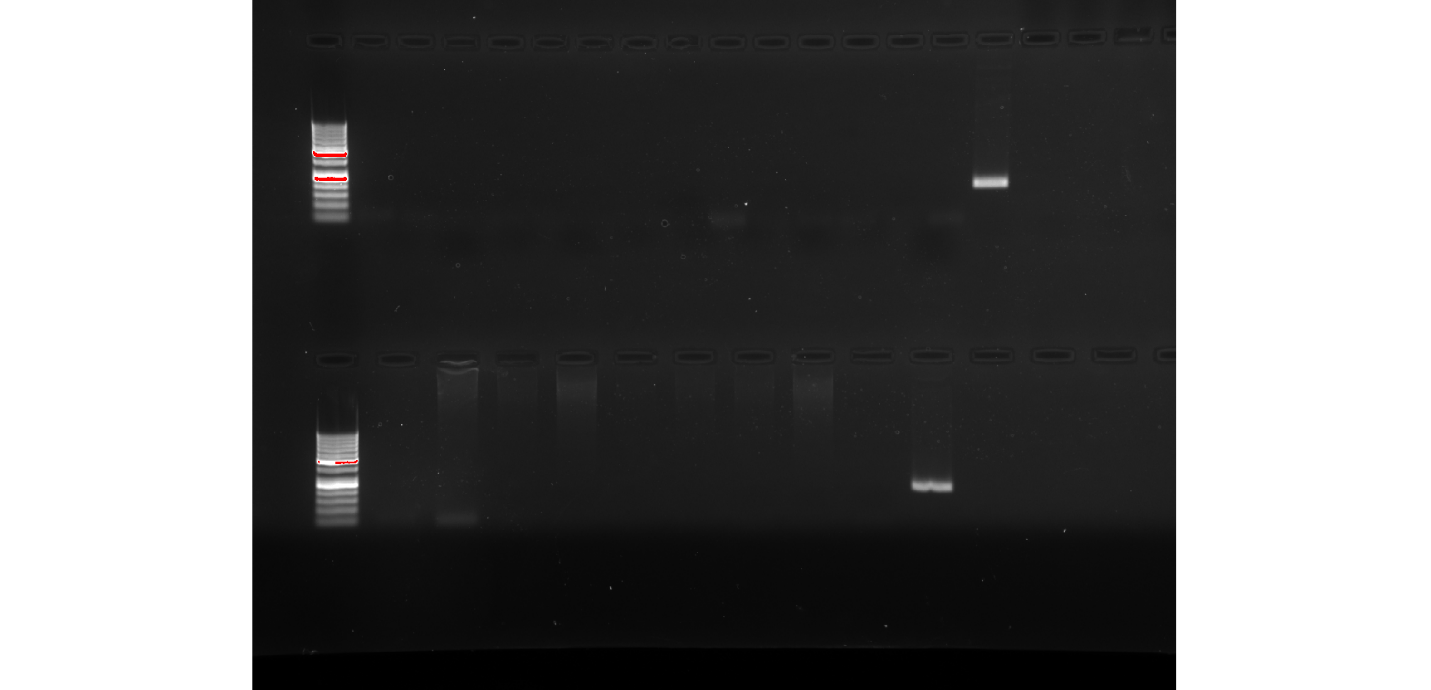


FP650 Negative controls top gel from left to right: 50 bp ladder, R5A, Y861, Y850, 4849A, R48, W6, W74, 891A, Y97A, 891B, 500A, FetB, Y868, 500B, Water, GFP Plasmid

Bottom Gel from left to right: 50bp ladder, 1462 COTY, 1477 COTY, 1767A COTY, 1767B COTY, 1555A COTY, 1763 COTY, 1237B COTY, Water, Empty, FP650 Plasmid
